# pSLIP: SVM based protein subcellular localization prediction using multiple physicochemical properties

**DOI:** 10.1186/1471-2105-6-152

**Published:** 2005-06-17

**Authors:** Deepak Sarda, Gek Huey Chua, Kuo-Bin Li, Arun Krishnan

**Affiliations:** 1 Bioinformatics Institute, 30, Biopolis Street, #07-01, Singapore – 138671

## Abstract

**Background:**

Protein subcellular localization is an important determinant of protein function and hence, reliable methods for prediction of localization are needed. A number of prediction algorithms have been developed based on amino acid compositions or on the N-terminal characteristics (signal peptides) of proteins. However, such approaches lead to a loss of contextual information. Moreover, where information about the physicochemical properties of amino acids has been used, the methods employed to exploit that information are less than optimal and could use the information more effectively.

**Results:**

In this paper, we propose a new algorithm called pSLIP which uses Support Vector Machines (SVMs) in conjunction with multiple physicochemical properties of amino acids to predict protein subcellular localization in eukaryotes across six different locations, namely, chloroplast, cytoplasmic, extracellular, mitochondrial, nuclear and plasma membrane. The algorithm was applied to the dataset provided by Park and Kanehisa and we obtained prediction accuracies for the different classes ranging from 87.7% – 97.0% with an overall accuracy of 93.1%.

**Conclusion:**

This study presents a physicochemical property based protein localization prediction algorithm. Unlike other algorithms, contextual information is preserved by dividing the protein sequences into clusters. The prediction accuracy shows an improvement over other algorithms based on various types of amino acid composition (single, pair and gapped pair). We have also implemented a web server to predict protein localization across the six classes (available at ).

## Background

One of the biggest challenges facing biologists today is the structural and functional classification and characterization of protein sequences. For example, in humans, the number of proteins for which the structures and functions are unknown makes up more than 40% of the total number of proteins. As a result, over the past couple of decades, extensive research has been done on trying to identify the structures and functions of proteins.

It is well known that the subcellular localization of proteins plays a crucial role in their functions [1]. A number of computational approaches have been developed over the years to predict the localization of proteins, including recent works like [2-12].

Initial efforts relied on amino acid compositions [13,14], the prediction of signal peptides [15-19] or a combination of both [20,21]. Later efforts were targeted at incorporating sequence order information (in the form of dipeptide compositions etc.) in the prediction algorithms [22-27].

There are drawbacks associated with all these methods. For example, prediction algorithms based on amino acid compositions suffer from the drawback that there is a loss of contextual information. As a result, sequences which are completely different in function and localization but that have a very similar amino acid composition would both be predicted as belonging to the same region of the cell. On the other hand, approaches that rely on predicting signal peptides can lead to inaccurate predictions when the signals are missing or only partially included [13].

Recent efforts have also focused on the use of physicochemical properties to predict subcellular localization of proteins [28,29]. Bhasin *et al*. [30] created an algorithm which was a hybrid of four different predictive methods. In addition to using amino acid compositions and dipeptide composition information, they also included 33 different physicochemical properties of amino acids, averaged over the entire protein. However such a globally averaged value again leads to a loss of contextual information. Bickmore *et al*. [31] studied the characteristics of the primary sequences of different proteins and concluded that motifs and domains are often shared amongst proteins co-localized within the same sub-nuclear compartment. Since the structure and hence the function of proteins is dictated by the different interacting physicochemical properties of the amino acids making up the protein, it would stand to reason that co-localized proteins must share some conservation in the different properties.

In this paper, we present a new algorithm called pSLIP: *Prediction of Subcellular Localization in Proteins*. We use multiple physicochemical properties of amino acids to obtain protein extracellular and subcellular localization predictions. A series of SVM based binary classifiers along with a new voting scheme enables us to obtain high prediction accuracies for six different localizations.

## Results and discussion

We implemented our algorithm on Park and Kanehisa's dataset [Table 1]. We divided the dataset into clusters based on sequence length and ran *N-fold cross validation (NF-CV) *tests for each of the protein clusters. The accuracies for each of these clusters were recorded and finally, these cluster accuracies were combined to produce overall accuracies. Table 2 lists them along with results from Park and Kanehisa's work [21] and Chou and Cai's work [8]. The protein subcellular localization method used in [21] is based on amino acid compositions. Chou and Cai's method [8] uses a hybrid algorithm called GO-FunD-PseAA [5] that combines gene ontology [32], functional domain decomposition [33] and pseudo-amino acid composition [26] for localization prediction.

**Table 1:** The number of proteins in the dataset. * These classes were not considered as they have too few proteins to achieve reliable training.

Subcellular Location	Number of entries
Chloroplast	671
Cytoplasmic	1241
Cytoskeleton*	40
Endoplasmic reticulum*	114
Extracellular	861
Golgi apparatus*	47
Lysosomal*	93
Mitochondrial	727
Nuclear	1932
Peroxisomal*	125
Plasma membrane	1674
Vacuolar*	54

Total	7579

**Table 2:** Sensitivity (sens) and Specificity (spec) (in %) on Park and Kanehisa's dataset [21]. First two columns show results from Park and Kanehisa's algorithm [21] obtained by 5-fold crossvalidation. The next column shows results from Chou and Cai's work [8] obtained using leave one out test. The last set of results are from our algorithm obtained using 5-fold and 10-fold crossvalidation.

Subcellular	P & K	P & K	Chou Cai	pSLIP			
Location	5-fold	5-fold	LOO-CV	5-fold		10-fold	
	sens	sens	sens	spec	sens	spec	sens

Chloroplast	72.3	70.3	93.9	89.9	84.8	93.5	92.4
Cytoplasmic	72.2	73.9	91.5	86.2	84.1	91.6	87.7
Cytoskeleton	58.5	59.8	80.0	-	-	-	-
ER	46.5	39.0	90.3	-	-	-	-
Extracellular	78.0	77.1	90.0	96.3	92.0	98.4	93.7
Golgi Apparatus	14.6	-	76.6	-	-	-	-
Lysosomal	61.8	62.4	92.5	-	-	-	-
Mitochondrial	57.4	53.5	83.6	75.9	86.8	85.7	93.5
Nuclear	89.6	89.0	95.3	89.4	90.4	93.1	93.1
Peroxisomal	25.6	-	82.4	-	-	-	-
Plasma Membrane	92.2	91.9	95.0	95.1	94.1	95.0	97.0
Vacuolar	25.0	-	66.7	-	-	-	-

TA	78.2	79.1	92.4	89.5		93.1	
LA	57.9	68.5	-	88.7		92.9	

The results reported by Park and Kanehisa are obtained after 5-fold cross validation testing. To ensure fairness in comparing results, we ran a 5-fold test on our algorithm. As is apparent from Table 2, our method provides good overall accuracy of 89.5% which is significantly higher than 78.2% and 79.1% obtained for the two different cases from Park and Kanehisa's paper. Even more interesting is the fact that the accuracies obtained by Park and Kanehisa are skewed towards those locations that have the most number of proteins in the dataset, viz., nuclear and plasma membrane. Total accuracies can sometimes present a misleading picture about the efficacy of a classification technique. Local accuracies, on the other hand can provide a more realistic view of classification efficiencies. We obtained a local accuracy of 88.7% which is only slightly less than the overall accuracy (89.5%) of the technique. On the other hand, the local accuracies obtained by Park and Kanehisa are significantly lower than the corresponding total accuracies (57.9% and 68.5% when compared with total accuracies of 78.2% and 79.1% respectively.)

Chou and Cai have used the *leave one out cross validation (LOO-CV) *test to assess the performance of their GO-FunD-PseAA predictor. Due to reasons described later, we've used only NF-CV tests. In order to make a reasonable comparison with their results, we did a 10-fold test which provides a good trade-off between bias and variance in test results. As results in Table 2 show, our algorithm performs as well as the GO-FunD-PseAA predictor and the obtained accuracy of 93.1% compares favorably with the 92.4% accuracy obtained by Chou and Cai. Although Chou and Cai's work tackles the harder problem of classifying over more subcellular locations than we do, the results do show the promise in the approach of using physicochemical properties for localization prediction.

Table 3 shows the classification performance for each of the individual clusters. We found that the prediction accuracies for each of the classes is largely uniform across the different clusters. However, for the cluster with base length of 450, the classification performance for all classes are uniformly lower than the accuracies obtained for the other clusters. This is probably due to the presence of sequences of lengths far greater than the base length of that cluster. The was because of an insufficient number of sequences of lengths greater than 1350 (in order to form a separate cluster of their own.) However, it is clear that if not for this aberration, the overall accuracies for this method would be higher.

**Table 3:** Cluster-wise Specificity (spec) and Sensitivity (sens) (in %) for pSLIP using 10-fold cross validation.

Base length	50		150		300		450		Overall	
	spec	sens	spec	sens	spec	sens	spec	sens	spec	sens

Chloroplast	99.3	97.9	95.9	93.0	97.3	89.1	83.0	90.2	93.5	92.4
Cytoplasmic	95.6	90.3	94.6	93.4	89.4	92.9	89.9	78.5	91.6	87.7
Extracellular	99.1	100	99.0	98.1	97.8	89.3	96.3	80.4	98.4	93.7
Mitochondrial	95.6	96.6	92.1	95.0	88.5	91.0	73.3	92.0	85.7	93.5
Nuclear	93.6	94.6	95.3	95.8	93.6	93.6	91.4	90.8	93.1	93.1
Plasma Memb	95.9	98.9	96.9	98.8	97.4	99.0	92.7	94.8	95.0	97.0

TA	96.4		95.9		93.8		89.3		93.1	
LA	96.4		95.7		92.5		87.8		92.9	

Cross validation experiments are frequently prone to an optimistic bias [34]. This occurs because the experimental setup can be such that the choice of the learning machine's parameters becomes dependent on the test data. We've tried to minimize the possible effect of this by using only a small subset (ninety sequences of each type) of the available sequences for parameter search, as described later in this paper. As a further experiment, we've also carried out an *independent dataset (ID) *test using the the eukaryotic sequences dataset developed by Reinhardt and Hubbard [14]. This dataset has also been widely used for subcellular localization studies. Instead of doing cross-validation testing on this dataset, we use the SVM classifiers generated by our method using Park and Kanehisa's dataset and predict the subcellular localization of all sequences in the Reinhardt and Hubbard dataset.

The results of this test, along with the results obtained by others using this dataset, are shown in Table 4. The first set of results are from Reinhardt and Hubbard's work [14] in which a neural network based classifier built upon amino acid composition as input is used. The next set of results are from Hua and Sun [13] who used an amino acid composition based SVM method. Esub8 [27] uses some sequence information along with amino acid composition for classification using SVM. ESLpred [30] is a hybrid model combining amino acid composition, dipeptide composition and average physicochemical properties as features for sequences that are then classified by SVM.

**Table 4:** Classification performance (sensitivity) (in %) on Reinhardt and Hubbard's dataset [14]; NF-CV: Results are given by N-fold cross validation. LOO-CV: Results are given by leave one out cross validation test. ID: Results are given by directly testing entire dataset, without any training on this dataset.

Subcellular	R&H	SubLoc	Esub8	ESLpred	pSLIP
Location	NF-CV	LOO-CV	LOO-CV	NF-CV	ID
Cytoplasmic	55	76.9	80	85.2	75.9
Extracellular	75	80	86.5	88.9	76.3
Mitochondrial	61	56.7	67.6	68.2	85.3
Nuclear	72	87.4	91.2	95.3	84.2

TA	66.0	79.4	84.14	88.0	81.0
LA	65.8	75.3	81.3	84.4	80.4

The results in Table 4 illustrate the importance of incorporating sequence order information in the classification method. The first two methods ignore order information entirely and we believe that their prediction accuracy suffers as a result of this. Furthermore, although the prediction accuracies of Esub8 and ESLpred are better than those of our method, it must be borne in mind that these results are from training and testing on the same dataset while our results are from training the classifiers on a different dataset. It must be noted however that the prediction accuracies for mitochondrial proteins, which are notoriously difficult to predict, are significantly higher using our method than any of the other methods (85.3% as compared to accuracies between 56% and 68.2% for the other methods).

The GO-FunD-PseAA predictor, whose classification performance on the Park and Kanehisa dataset is shown in Table 2, has also been tested on the Reinhardt and Hubbard dataset. The predictor performs well on this dataset too and yields the highest total accuracy of 92.9% [5] using the rigorous leave one out cross validation test. However, we could not include these results in Table 4 since the results in [5] do not provide a subcellular location-wise breakdown of prediction performance.

We have implemented our algorithm for predicting subcellular localizations as a web server which can be accessed at .

## Conclusion

Protein subcellular localization has been an active area of research due to the important role it plays in indicating, if not determining, protein function. A number of efforts have previously used amino acid compositions as well as limited sequence order information in order to predict protein localization. In this work, we have developed a novel approach based on using multiple physicochemical properties. In order to use sequence order information, we divide the set of proteins into four different clusters based on their lengths. Within each cluster, proteins are mapped onto the lowest length in that cluster (50, 150, 300 and 450 for the four clusters).

We then developed multiple binary classifiers for each cluster. For each protein, the output from each binary classifier was encoded as a binary bit sequence to form a meta-dataset. To predict the localization of a query protein, a similar binary sequence was generated based on the outputs of the different binary classifiers and the nearest neighbor to this protein was sought in the meta-dataset.

We obtained significantly higher classification accuracies (93.1% overall and 92.1% local) for the Park and Kanehisa dataset. The prediction accuracies obtained for mitochondrial and extracellular proteins in particular are among the highest that have been achieved so far.

The clustered approach was chosen to not only be able to include sequence order information beyond that of di-, tri-and tetra-peptide information but also to mitigate the effects of over-averaging. One of the problems we encountered was the small number of proteins of length greater than 1350. As a result, these were averaged down to a base length of 450 leading to a drop in accuracies for the 450 cluster. Obviously larger datasets, with more representative samples in the length range greater than 1350 might yield greater accuracies.

## Methods

### Dataset

We used the protein sequences dataset^1^created by Park and Kanehisa [21]. The dataset consists of 7579 eukaryotic proteins drawn from the SWISS-PROT database and classified into twelve subcellular locations. The protein sequences were classified based on the keywords found in the CC (comments or notes) and OC (organism classification) fields of SWISS-PROT. Proteins annotated with multiple subcellular locations were not included in the dataset. Further, proteins containing B, X or Z in the amino acid sequence were excluded from the dataset. Finally, proteins with high sequence similarity (greater than 80%) were not chosen for inclusion.

Table 1 summarizes the number of sequences in each of the twelve subcellular locations. For some of the locations such as cytoskeleton, there were too few sample sequences to achieve reliable training accuracies using SVM, the machine learning algorithm used in this work. Hence, we considered only sequences of type: chloroplast, cytoplasmic, extracellular, mitochondrial, nuclear and plasma membrane resulting in a dataset with 7106 eukaryotic protein sequences.

### Support vector machine

The concept of Support Vector Machines (SVM) was first introduced by Vapnik [35,36] and in recent times, the SVM approach has been used extensively in the areas of classification and regression. SVM is a learning algorithm which, upon training with a set of positively and negatively labeled samples, produces a classifier that can then be used to identify the correct label for unlabeled samples. SVM builds a classifier by constructing an optimal hyperplane that divides the positively and the negative labeled samples with the maximum margin of separation. Each sample is described by a feature vector. Typically, training samples are not linearly separable. Hence, the feature vectors of all training samples are first mapped to a higher dimensional space *H *and an optimal dividing hyperplane is sought in this space.

The SVM algorithm requires the solving of a quadratic optimization problem. To simplify the problem, SVM does not explicitly map the feature vectors of all the samples to the space *H*. Instead, mapping is done implicitly by defining a kernel function  between two samples with feature vectors  and  as:



where Φ is the mapping to the space *H*.

For a detailed description of the mathematics behind SVM, we refer the reader to an article by Burges [37]. For the present study, we used the SVM*light *package (version 6.01) created by Joachims [38]. The package is available online^2 ^and is free for scientific use.

### Multi class SVM

A multi-class classification problem, such as the subcellular localization problem, is typically solved by reducing the multi-class problem into a series of binary classification problems. In the method employed here, called the 1-vs-1 method, a binary classifier is constructed for each pair of classes. Thus, a *c*-class problem is transformed into several two-class problems: one for each pair of classes (*i*, *j*), 1 ≤ *i*, *j *≤ *c*, *i *≠ *j*. We use the notation  to refer to both the binary classification problem of separating samples of classes *i *and *j *as well as the SVM classifier which is used to solve this problem. The classifier for the two-class problem  is trained with samples of classes *i *and *j*, ignoring samples from all other classes.

Since SVM is a symmetric learning algorithm, the classifier  is the same as the classifier . Thus, for the purpose of classification, it is sufficient if we consider only those classifiers  for which *i*<*j* Hence, we only construct classifiers for each pair of classes (*i*, *j*), 1 ≤ *i*, *j *≤ *c*, *i *<*j *and there are *c *(*c *- 1) /2 such classifiers. To illustrate, if a classification problem involved three classes *a*, *b *and *c*, the 1-vs-1 method would require construction of the binary classifiers  and .

We term those classifiers which are trained to differentiate the true class of the test sample from other classes as *relevant *classifiers while the remaining classifiers are termed *irrelevant *classifiers. For example, using the three class example cited above, if the (as yet unknown) true class of a test sample is *a*, then the relevant classifiers would be  and  while the classifier  would be irrelevant.

An unlabeled test sample is tested against all the *c *(*c *- 1) /2 binary classifiers and the predictions from each of these classifiers are combined by some algorithm to assign a class label to the sample. The design of the combining algorithm should be such that the predictions from the relevant classifiers gain precedence over those from the irrelevant classifiers. A simple *voting scheme *is one such algorithm that has been used earlier [27]. In this scheme, a vote is assigned to a class every time a classifier predicts that the test sample belongs to that class. The class with the maximum number of votes is deemed to be the true class of the sample.

The prediction performance of the voting scheme approach relies on the assumption that the relevant classifiers for the unlabeled sample perform very well and the number of votes they cast in favor of the true class outnumber the number of votes obtained by any other class from the irrelevant classifiers. In practice, we found this not to be the case. Some of the relevant classifiers performed poorly and a wrong class frequently got the highest number of votes by virtue of many irrelevant classifiers voting for it.

**Figure 1:**
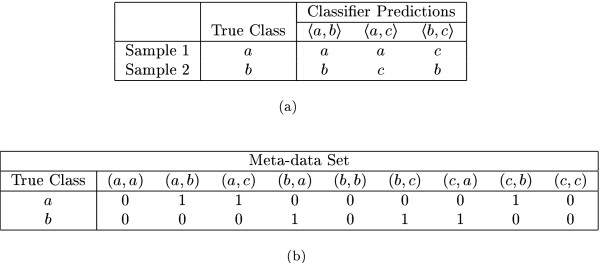
**Meta-data set construction. **This figure shows, using two samples, how the meta-data set is constructed for a three class problem. Figure 1(a) lists the predictions obtained by testing the two samples against all the binary classifiers. Figure 1(b) lists the bit sequences corresponding to the obtained predictions.

To solve this problem with combining classifier predictions, we replaced the voting scheme with a new classifier, called the *meta-classifier*. Described in [39], the meta-classifier works as follows: The set of all the two-class classifiers described above is first built using the available training samples. After this, each of the training samples is tested against all the binary classifiers (Figure 1(a)) and the class predictions are encoded in a binary (0 or 1) bit sequence (Figure 1(b)). We choose a bit sequence of length *c*^2 ^with one bit for each possible combination . All the bits in the sequence are initialized to zero. If a binary classifier  predicts the training sample to belong to class *p*, then the bit corresponding to the position (*p,q*) is set to 1; if the prediction is for class *q*, then the bit corresponding to the position (*q,p*) is set to 1.

Thus, after testing a sample with all the classifiers, we get an encoded representation of the classifier predictions in the form of a bit sequence. Since this sample is a training sample, it's true class (or label) is known. The true class and the bit sequence together constitute a meta-data instance derived from the training sample. The collection of all such meta-data instances derived from all the training samples is termed the *meta-data set*. Figure 1 provides an illustration of this process.

**Figure 2:**
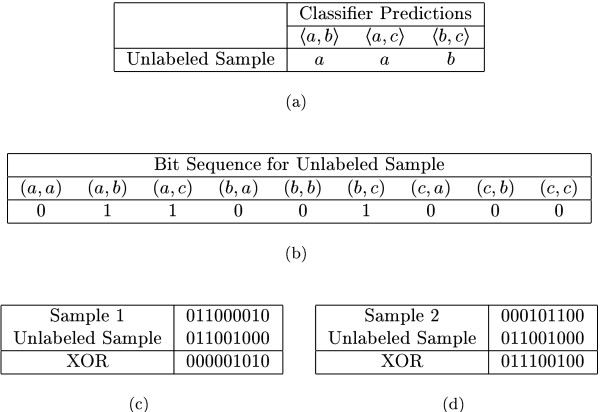
**Meta-classification. **The unlabeled sample is first tested against all the binary classifiers and Figure 2(a) shows the predictions obtained after such a test. Next, its bit sequence representation is constructed [Figure 2(b)]. The XOR operation is performed between this bit sequence and each of the sequences from the meta-data set [Figures 2(c) & 2(d)]. The distance between samples is the number of 1-bits in the XOR operation result. Since the distance of the unlabeled sample from sample 1 is less than that from sample 2, the unlabeled sample is assigned the label of sample 1, i.e. it is assigned to the class *a*.

When an unlabeled test sample is presented for classification, it is first tested against all the binary classifiers (Figure 2(a)) and the class predictions are encoded in a bit sequence (Figure 2(b)) as explained above. Then, we seek an instance in the meta-data set whose bit sequence most closely matches this test sample's bit sequence. To search for such a matching sequence, we use a nearest neighbor approach with the distance between two sequences being the number of bits in which they differ. The *Exclusive OR *(XOR) operator can be used to count the number of differing bits. Table 5 shows the truth table for the XOR operator. When we carry out a XOR operation between the test bit sequence and a sequence from the meta-data set, we get another bit sequence. The number of 1-bits in this resulting sequence gives a measure of distance with a higher number implying greater distance between the sequences. Figure 2 describes this procedure with an example. This distance calculation method is similar to the *hamming decoding *method described in [40].

**Table 5:** Truth table for the Exclusive OR (XOR) operator. Thus, for example, 101 XOR 011 would be 110.

XOR	0	1
0	0	1
1	1	0

After the sequence from the meta-data set that is closest to the test sequence is identified, we assign its known class label to the test sample. In case multiple (equidistant) sequences are found after the nearest neighbor search, resulting in a tie between two or more classes, we pick that particular class which would have got the maximum votes of the tied classes; the votes being counted according to the voting scheme described earlier. By implementing this meta-classification approach, we found an improvement in accuracy of 10% – 15% over the voting method.

### Feature vectors

Many previous efforts have used amino acid composition as the feature to determine protein subcellular localization. In these efforts, the feature vector corresponding to an amino acid sequence is typically a 20-dimensional vector with each element of the vector representing the frequency of occurrence of an amino acid in that particular sequence. As highlighted earlier, this approach leads to a complete loss of sequence order information. On the other hand, the averaging of physicochemical properties over the entire length of the protein sequence also results in a loss of sequence order information. We believe that sequences that are co-localized must share some similarity across certain physicochemical properties, regardless of their length.

To overcome the shortcomings of earlier efforts, we employ a novel method of building feature vectors which is based on an idea first proposed in [41]. Consider an amino acid sequence of length *L*. Suppose we wish to use *M *different physicochemical properties in the feature representation of the amino acid sequence. Corresponding to each amino acid *i*, we build *property vectors * (1,..., 20) where  (1,..., *M*) is the vector of normalized values of the *M *physicochemical properties for the amino acid *i*. Then, for the sequence of length *L*, we concatenate the property vectors of each of the amino acids in the sequence in succession to get a vector of dimension *L *× *M*.



where  is one of  depending upon which amino acid *i *is present at location *k *in the sequence.

While this method allows us to build feature vectors using any number of desired physicochemical properties, it results in vectors whose dimension is a function of the length of the amino acid sequence. One of the problems with using physicochemical properties averaged over the entire sequence length, in localization prediction efforts so far, has been the difference in lengths of the different protein sequences. Since SVM requires equal length feature vectors, this has always been a deterrent to utilizing sequence order information. Hence, we apply a local averaging process to scale all generated feature vectors to a standard dimension.

Suppose we take our standard dimension to be *K *and wish to construct a feature vector of this dimension for a protein sequence of length *L*. For now, let's assume that we are building amino acid property vectors using just one physicochemical property. So the feature vector , obtained by concatenating property vectors as described above, will also be of dimension *L*. We then sequentially group the feature vector's elements such that we end up with *K *groups or partitions of the vector. Withing each part, we take the average values of the constituent elements and then build a vector out of these averaged values. This operation is like constructing a mapping and can be thought of conceptually as reducing the protein sequence of length *L *to a standard length *l *and here *l *= *K*.

If we used *M *physicochemical properties instead of one, a conceptual scaling of a protein sequence of length *L *to a standard length *l *is equivalent to a scaling of  (of length *L *× *M*) to a standard dimension of *K *= *l × M*. To do this operation, we partition the amino acid sequence of length *L *into *l *nearly equal parts and then take the average value of the property vectors within these partitions.

**Figure 3:**
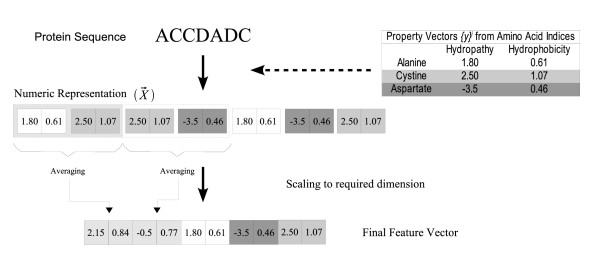
**Feature vector construction. **In this example, the physicochemical properties chosen are *hydropathy *and *hydrophobicity*. Their property vectors  are concatenated in the order of occurrence of the residues in the sample protein sequence. The  vector thus obtained is scaled by averaging to the required target dimension.

Figure 3 shows an example where the feature vector for an amino acid sequence of length *L *= 6 is constructed. The example uses *M *= 2 physicochemical properties and the target standard dimension is of length *K *= 10.

There is a loss of sequence order information due to this averaging process and this loss is significant when scaling down proteins of very long lengths to a much shorter length. To minimize this information loss, we divided our dataset into clusters defining a base length for each cluster. This base length is equivalent to the conceptual protein sequence standard length *l*. Within each cluster, no sequence has a length less than *l *and the length of the longest sequence is no greater than three times *l*. Initially, we built clusters using the base lengths as (10, 30, 90, 270, 810, 2430) but this resulted in an uneven population distribution of sequences across clusters that caused problems in the SVM training stage. We adjusted the cluster sizes and finally chose the base lengths as (50,150, 300, 450). For sequences of length less than fifty, we extended their length to fifty by suitably repeating the residues. For example, if the sequence *AMKMSF *of length six needs to be scaled to a length of ten, we would repeat residues to get *AAMMKKMMSF*.

### Parameter selection

Once the set of sequence clusters is built, we treat each of these clusters as a separate multi-class SVM problem. For each of the clusters, we build a set of binary classifiers as explained earlier. For the SVM algorithm itself, the kernel  is to be defined first. We chose the widely used *Radial Basis Function (RBF) *kernel which is defined as:



Further, the SVM optimization model employs a regularization parameter *C *which controls the trade-off between the margin of separation (between positive and negatively labeled samples) and the error in classification. Thus, the process of parameter selection for the classification problem repeats over the set of binary classifiers per cluster and then for each of the clusters.

There are three parameters that need to be ascertained:

1. The set of *M *physicochemical properties to represent the amino acid sequences

2. The value of γ for the kernel function

3. The value of the regularization parameter *C*

For the set of physicochemical properties, we used the Amino Acid index database [42] available at . An amino acid index is a set of 20 numerical values representing any of the different physicochemical properties of amino acids. This database currently contains 484 such indices.

**Figure 4:**
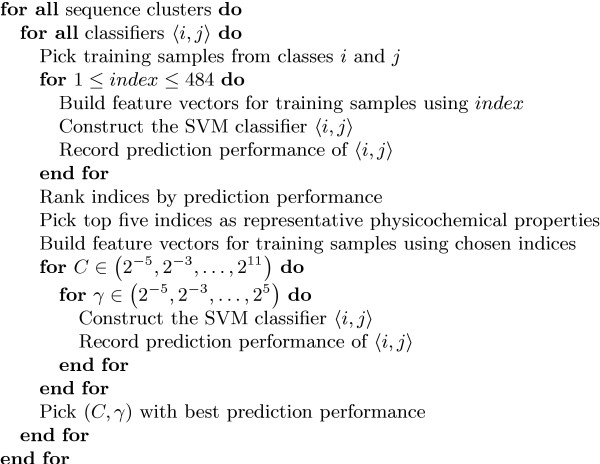
**Parameter search. **This shows the approach taken to find the best parameters for the different possible combinations of sequence clusters and classifiers.

The process of selecting the parameters is carried out using the approach shown in Figure 4 which essentially involves doing a parameter space search for the different possible combinations of sequence clusters, classifiers and parameters. We first determine the prediction performances that can be obtained by the different classifiers in the different clusters by building feature vectors with each of the 484 indices taken one at a time. During this search over the indices, we let SVM*light *assign default values to *C *and γ. Once this search is done, we pick the top five best performing indices to be the representative physicochemical properties (for that particular combination of cluster and classifier.)

We then build feature vectors using these top five indices and do a search over the *C *and γ space looking for the best performing combination of these parameters. At the end of this parameter search, we obtain the best combination of amino acid indices, *C *and γ for each classifier in each sequence cluster. We then look at which sequence cluster achieved the best overall prediction performance and pick the set of best parameters for classifiers in that cluster as the best set for all the clusters.

The set of top five amino acid indices for each of the classifiers as found using this parameter search for the Park and Kanehisa dataset have been provided [see Additional file 1].

### Validation

To assess the prediction performance of the proposed algorithm, a cross-validation test must be performed. The three methods most often used for cross-validation are the *independent dataset (ID) *test, the *leave one out cross validation (LOO-CV) *test and the *N-fold cross validation (NF-CV) *test [43]. Of the three, the LOO-CV test is considered to be the most rigorous and objective [44]. Although bias-free, this test is very computationally demanding and is often impractical for large datasets. Further, it suffers from possibly high variance in results depending on the composition of the dataset and the characteristics of the classifier. The NF-CV test provides a bias-free estimate of the accuracy [45] at a much reduced computational cost and is considered an acceptable test for evaluating prediction performance of an algorithm [46].

In NF-CV tests, the dataset is divided into *N *parts with approximately equal number of samples in each part. The learning machine is trained with samples from *N *- 1 parts while the *Nth *part is used as testing set to calculate classification accuracies. The learning-testing process is repeated *N *times until each part has been used as a testing set once.

Since the number of protein sequence samples in each class are all different, it is obvious that during training phase of a binary classifier, the number of training samples in the two classes will not be equal. If the SVM is trained on these unequally sized sets, the resulting classifier will be inherently biased toward the more populous class; it is more likely to predict a test sample to belong to that class. The greater the disparity in populations between the two classes, the more pronounced the bias is. It is difficult to prevent this bias in the training stage without adjusting more parameters on a per classifier level. To prevent this problem, we reduce the training set to an equisized set by randomly selecting *m *samples from the larger set; *m *being the size of the smaller set.

To quantify the performance of our proposed algorithm, we use the widely used measures of *Specificity *and *Sensitivity*. Let *N *be the total number of proteins in the testing dataset and let *k *be the number of subcellular locations (classes). Let  be the number of proteins of class *i *classified by the algorithm as belonging to class *j*.

The specificity, also called *precision*, for class *i *measures how many of the proteins classified as belonging to class *i *truly belong to that class.



The sensitivity, also called *recall*, for class *i *measures how many of the proteins truly belonging to class *i *were correctly classified as belonging to that class.



We further define *Total Accuracy *to measure how many proteins overall were correctly classified.



It is expected that the most populous classes will dominate the total accuracy measure and a classifier biased towards those classes will perform well according to this measure, even if the prediction performance for the smaller sized classes is not good. Hence, we consider another measure termed *Location Accuracy *and defined as:



Location accuracy reveals any poor performance by an individual classifier by providing a measure of how well the classification works for each class of proteins.

The definitions of Total Accuracy and Local Accuracy used here are equivalent to those used by Park and Kanehisa [21].

## Authors' contributions

DS and GC designed and implemented the pSLIP algorithm along with the voting scheme. KL and AK conceived the study, participated in its design and supervised the process. AK and DS drafted the manuscript. All authors read and approved the final manuscript.

## Supplementary Material

Additional File 1 This file lists the top five amino acid indices found by parameter search for each of the binary classifiers. Click here for file
