# Supplementary material for: pSLIP: SVM based protein subcellular localization prediction using multiple physicochemical properties
Source: BMC Bioinformatics. 2005 Jun 17;6:152. doi: 10.1186/1471-2105-6-152 (PMC1182350; doi:10.1186/1471-2105-6-152)
Supplement: Additional File 1 — This file lists the top five amino acid indices found by parameter search for each of the binary classifiers. [file 1471-2105-6-152-S1.pdf]

Supplementary Material

Deepak Sarda, Gek Huey Chua, Kuo-Bin Li, Francis Tang and Arun Krishnan*

May 24, 2005

*To whom correspondence should be addressed.

Classifier	Best Indices				
(0, 1)	FUKS010108	CEDJ970101	KRIW710101	NAKH920107	JUKT750101
(0, 2)	PONP800103	BUNA790103	LEVM760102	PLIV810101	OOBM770101
(0, 3)	FUKS010105	BUNA790103	NAKH920104	CEDJ970101	NAKH920106
(0, 4)	KRIW790101	PLIV810101	PONP800103	LEVM760102	OOBM770101
(0, 5)	JUKT750101	KRIW790101	KRIW710101	FAUJ880101	OOBM770101
(1, 2)	LEVM760102	FAUJ880107	TAKK010101	SIMZ760101	ZIMJ680105
(1, 3)	CIDH920104	QIAN880120	TAKK010101	PONP800104	ZIMJ680105
(1, 4)	TANS770110	PONP800103	PALJ810104	OOBM770101	ZIMJ680105
(1, 5)	NAKH900112	PONP800103	FAUJ880101	PLIV810101	OOBM770101
(2, 3)	NAKH900112	NAKH900109	TAKK010101	NAKH900105	OOBM770101
(2, 4)	PONP800103	NAKH900102	PLIV810101	OOBM770101	LEVM760102
(2, 5)	KRIW710101	PLIV810101	JUKT750101	OOBM850105	OOBM770101
(3, 4)	JUKT750101	ZIMJ680105	NAKH920102	PONP800103	KRIW790101
(3, 5)	JUKT750101	KRIW710101	KRIW790101	PLIV810101	OOBM770101
(4, 5)	JUKT750101	PONP800103	KRIW790101	PLIV810101	OOBM770101

Table 1: The top five indices chosen for each binary classifier: 0: chloroplast 1: cytoplasmic 2: extracellular 3: mitochondrial 4: nuclear 5: plasma membrane

AAIndex	Description
ANDN920101	alpha-CH chemical shifts (Andersen et al., 1992)
ARGP820101	Hydrophobicity index (Argos et al., 1982)
ARGP820102	Signal sequence helical potential (Argos et al., 1982)
ARGP820103	Membrane-buried preference parameters (Argos et al., 1982)
BEGF750101	Conformational parameter of inner helix (Beghin-Dirkx, 1975)
BEGF750102	Conformational parameter of beta-structure (Beghin-Dirkx, 1975)
BEGF750103	Conformational parameter of beta-turn (Beghin-Dirkx, 1975)
BHAR880101	Average flexibility indices (Bhaskaran-Ponnuswamy, 1988)
BIGC670101	Residue volume (Bigelow, 1967)
BIOV880101	Information value for accessibility; average fraction 35% (Biou et al., 1988)
BIOV880102	Information value for accessibility; average fraction 23% (Biou et al., 1988)
BROC820101	Retention coefficient in TFA (Browne et al., 1982)
BROC820102	Retention coefficient in HFBA (Browne et al., 1982)
BULH740101	Transfer free energy to surface (Bull-Breese, 1974)
BULH740102	Apparent partial specific volume (Bull-Breese, 1974)
BUNA790101	alpha-NH chemical shifts (Bundi-Wuthrich, 1979)
BUNA790102	alpha-CH chemical shifts (Bundi-Wuthrich, 1979)
BUNA790103	Spin-spin coupling constants 3JHalpha-NH (Bundi-Wuthrich, 1979)
BURA740101	Normalized frequency of alpha-helix (Burgess et al., 1974)
BURA740102	Normalized frequency of extended structure (Burgess et al., 1974)
CHAM810101	Steric parameter (Charton, 1981)
CHAM820101	Polarizability parameter (Charton-Charton, 1982)
CHAM820102	Free energy of solution in water, kcal/mole (Charton-Charton, 1982)
CHAM830101	The Chou-Fasman parameter of the coil conformation (Charton-Charton, 1983)
CHAM830102	A parameter defined from the residuals obtained from the best correlation of the Chou-Fasman parameter of beta-sheet (Charton-Charton, 1983)
CHAM830103	The number of atoms in the side chain labelled 1+1 (Charton-Charton, 1983)
CHAM830104	The number of atoms in the side chain labelled 2+1 (Charton-Charton, 1983)
CHAM830105	The number of atoms in the side chain labelled 3+1 (Charton-Charton, 1983)
CHAM830106	The number of bonds in the longest chain (Charton-Charton, 1983)
CHAM830107	A parameter of charge transfer capability (Charton-Charton, 1983)
CHAM830108	A parameter of charge transfer donor capability (Charton-Charton, 1983)
CHOC750101	Average volume of buried residue (Chothia, 1975)
CHOC760101	Residue accessible surface area in tripeptide (Chothia, 1976)
CHOC760102	Residue accessible surface area in folded protein (Chothia, 1976)
CHOC760103	Proportion of residues 95% buried (Chothia, 1976)
CHOC760104	Proportion of residues 100% buried (Chothia, 1976)
CHOP780101	Normalized frequency of beta-turn (Chou-Fasman, 1978a)

AAIndex	Description
CHOP780201	Normalized frequency of alpha-helix (Chou-Fasman, 1978b)
CHOP780202	Normalized frequency of beta-sheet (Chou-Fasman, 1978b)
CHOP780203	Normalized frequency of beta-turn (Chou-Fasman, 1978b)
CHOP780204	Normalized frequency of N-terminal helix (Chou-Fasman, 1978b)
CHOP780205	Normalized frequency of C-terminal helix (Chou-Fasman, 1978b)
CHOP780206	Normalized frequency of N-terminal non helical region (Chou-Fasman, 1978b)
CHOP780207	Normalized frequency of C-terminal non helical region (Chou-Fasman, 1978b)
CHOP780208	Normalized frequency of N-terminal beta-sheet (Chou-Fasman, 1978b)
CHOP780209	Normalized frequency of C-terminal beta-sheet (Chou-Fasman, 1978b)
CHOP780210	Normalized frequency of N-terminal non beta region (Chou-Fasman, 1978b)
CHOP780211	Normalized frequency of C-terminal non beta region (Chou-Fasman, 1978b)
CHOP780212	Frequency of the 1st residue in turn (Chou-Fasman, 1978b)
CHOP780213	Frequency of the 2nd residue in turn (Chou-Fasman, 1978b)
CHOP780214	Frequency of the 3rd residue in turn (Chou-Fasman, 1978b)
CHOP780215	Frequency of the 4th residue in turn (Chou-Fasman, 1978b)
CHOP780216	Normalized frequency of the 2nd and 3rd residues in turn (Chou-Fasman, 1978b)
CIDH920101	Normalized hydrophobicity scales for alpha-proteins (Cid et al., 1992)
CIDH920102	Normalized hydrophobicity scales for beta-proteins (Cid et al., 1992)
CIDH920103	Normalized hydrophobicity scales for alpha+beta-proteins (Cid et al., 1992)
CIDH920104	Normalized hydrophobicity scales for alpha/beta-proteins (Cid et al., 1992)
CIDH920105	Normalized average hydrophobicity scales (Cid et al., 1992)
COHE430101	Partial specific volume (Cohn-Edsall, 1943)
CRAJ730101	Normalized frequency of middle helix (Crawford et al., 1973)
CRAJ730102	Normalized frequency of beta-sheet (Crawford et al., 1973)
CRAJ730103	Normalized frequency of turn (Crawford et al., 1973)
DAWD720101	Size (Dawson, 1972)
DAYM780101	Amino acid composition (Dayhoff et al., 1978a)
DAYM780201	Relative mutability (Dayhoff et al., 1978b)
DESM900101	Membrane preference for cytochrome b: MPH89 (Degli Esposti et al., 1990)
DESM900102	Average membrane preference: AMP07 (Degli Esposti et al., 1990)
EISD840101	Consensus normalized hydrophobicity scale (Eisenberg, 1984)
EISD860101	Solvation free energy (Eisenberg-McLachlan, 1986)
EISD860102	Atom-based hydrophobic moment (Eisenberg-McLachlan, 1986)
EISD860103	Direction of hydrophobic moment (Eisenberg-McLachlan, 1986)
FASG760101	Molecular weight (Fasman, 1976)
FASG760102	Melting point (Fasman, 1976)
FASG760103	Optical rotation (Fasman, 1976)
FASG760104	pK-N (Fasman, 1976)
FASG760105	pK-C (Fasman, 1976)

AAIndex	Description
FAUJ830101	Hydrophobic parameter pi (Fauchere-Pliska, 1983)
FAUJ880101	Graph shape index (Fauchere et al., 1988)
FAUJ880102	Smoothed upsilon steric parameter (Fauchere et al., 1988)
FAUJ880103	Normalized van der Waals volume (Fauchere et al., 1988)
FAUJ880104	STERIMOL length of the side chain (Fauchere et al., 1988)
FAUJ880105	STERIMOL minimum width of the side chain (Fauchere et al., 1988)
FAUJ880106	STERIMOL maximum width of the side chain (Fauchere et al., 1988)
FAUJ880107	N.m.r. chemical shift of alpha-carbon (Fauchere et al., 1988)
FAUJ880108	Localized electrical effect (Fauchere et al., 1988)
FAUJ880109	Number of hydrogen bond donors (Fauchere et al., 1988)
FAUJ880110	Number of full nonbonding orbitals (Fauchere et al., 1988)
FAUJ880111	Positive charge (Fauchere et al., 1988)
FAUJ880112	Negative charge (Fauchere et al., 1988)
FAUJ880113	pK-a(RCOOH) (Fauchere et al., 1988)
FINA770101	Helix-coil equilibrium constant (Finkelstein-Ptitsyn, 1977)
FINA910101	Helix initiation parameter at posision i-1 (Finkelstein et al., 1991)
FINA910102	Helix initiation parameter at posision i,i+1,i+2 (Finkelstein et al., 1991)
FINA910103	Helix termination parameter at posision j-2,j-1,j (Finkelstein et al., 1991)
FINA910104	Helix termination parameter at posision j+1 (Finkelstein et al., 1991)
GARJ730101	Partition coefficient (Garel et al., 1973)
GEIM800101	Alpha-helix indices (Geisow-Roberts, 1980)
GEIM800102	Alpha-helix indices for alpha-proteins (Geisow-Roberts, 1980)
GEIM800103	Alpha-helix indices for beta-proteins (Geisow-Roberts, 1980)
GEIM800104	Alpha-helix indices for alpha/beta-proteins (Geisow-Roberts, 1980)
GEIM800105	Beta-strand indices (Geisow-Roberts, 1980)
GEIM800106	Beta-strand indices for beta-proteins (Geisow-Roberts, 1980)
GEIM800107	Beta-strand indices for alpha/beta-proteins (Geisow-Roberts, 1980)
GEIM800108	Aperiodic indices (Geisow-Roberts, 1980)
GEIM800109	Aperiodic indices for alpha-proteins (Geisow-Roberts, 1980)
GEIM800110	Aperiodic indices for beta-proteins (Geisow-Roberts, 1980)
GEIM800111	Aperiodic indices for alpha/beta-proteins (Geisow-Roberts, 1980)
GOLD730101	Hydrophobicity factor (Goldsack-Chalifoux, 1973)
GOLD730102	Residue volume (Goldsack-Chalifoux, 1973)
GRAR740101	Composition (Grantham, 1974)
GRAR740102	Polarity (Grantham, 1974)
GRAR740103	Volume (Grantham, 1974)
GUYH850101	Partition energy (Guy, 1985)
HOPA770101	Hydration number (Hopfinger, 1971), Cited by Charton-Charton (1982)
HOPT810101	Hydrophilicity value (Hopp-Woods, 1981)

AAIndex	Description
HUTJ700101	Heat capacity (Hutchens, 1970)
HUTJ700102	Absolute entropy (Hutchens, 1970)
HUTJ700103	Entropy of formation (Hutchens, 1970)
ISOY800101	Normalized relative frequency of alpha-helix (Isogai et al., 1980)
ISOY800102	Normalized relative frequency of extended structure (Isogai et al., 1980)
ISOY800103	Normalized relative frequency of bend (Isogai et al., 1980)
ISOY800104	Normalized relative frequency of bend R (Isogai et al., 1980)
ISOY800105	Normalized relative frequency of bend S (Isogai et al., 1980)
ISOY800106	Normalized relative frequency of helix end (Isogai et al., 1980)
ISOY800107	Normalized relative frequency of double bend (Isogai et al., 1980)
ISOY800108	Normalized relative frequency of coil (Isogai et al., 1980)
JANJ780101	Average accessible surface area (Janin et al., 1978)
JANJ780102	Percentage of buried residues (Janin et al., 1978)
JANJ780103	Percentage of exposed residues (Janin et al., 1978)
JANJ790101	Ratio of buried and accessible molar fractions (Janin, 1979)
JANJ790102	Transfer free energy (Janin, 1979)
JOND750101	Hydrophobicity (Jones, 1975)
JOND750102	pK (-COOH) (Jones, 1975)
JOND920101	Relative frequency of occurrence (Jones et al., 1992)
JOND920102	Relative mutability (Jones et al., 1992)
JUKT750101	Amino acid distribution (Jukes et al., 1975)
JUNJ780101	Sequence frequency (Jungck, 1978)
KANM800101	Average relative probability of helix (Kanehisa-Tsong, 1980)
KANM800102	Average relative probability of beta-sheet (Kanehisa-Tsong, 1980)
KANM800103	Average relative probability of inner helix (Kanehisa-Tsong, 1980)
KANM800104	Average relative probability of inner beta-sheet (Kanehisa-Tsong, 1980)
KARP850101	Flexibility parameter for no rigid neighbors (Karplus-Schulz, 1985)
KARP850102	Flexibility parameter for one rigid neighbor (Karplus-Schulz, 1985)
KARP850103	Flexibility parameter for two rigid neighbors (Karplus-Schulz, 1985)
KHAG800101	The Kerr-constant increments (Khanarian-Moore, 1980)
KLEP840101	Net charge (Klein et al., 1984)
KRIW710101	Side chain interaction parameter (Krigbaum-Rubin, 1971)
KRIW790101	Side chain interaction parameter (Krigbaum-Komoriya, 1979)
KRIW790102	Fraction of site occupied by water (Krigbaum-Komoriya, 1979)
KRIW790103	Side chain volume (Krigbaum-Komoriya, 1979)
KYTJ820101	Hydropathy index (Kyte-Doolittle, 1982)
LAWE840101	Transfer free energy, CHP/water (Lawson et al., 1984)
LEVM760101	Hydrophobic parameter (Levitt, 1976)
LEVM760102	Distance between C-alpha and centroid of side chain (Levitt, 1976)

AAIndex	Description
LEVM760103	Side chain angle theta(AAR) (Levitt, 1976)
LEVM760104	Side chain torsion angle phi(AAAR) (Levitt, 1976)
LEVM760105	Radius of gyration of side chain (Levitt, 1976)
LEVM760106	van der Waals parameter R0 (Levitt, 1976)
LEVM760107	van der Waals parameter epsilon (Levitt, 1976)
LEVM780101	Normalized frequency of alpha-helix, with weights (Levitt, 1978)
LEVM780102	Normalized frequency of beta-sheet, with weights (Levitt, 1978)
LEVM780103	Normalized frequency of reverse turn, with weights (Levitt, 1978)
LEVM780104	Normalized frequency of alpha-helix, unweighted (Levitt, 1978)
LEVM780105	Normalized frequency of beta-sheet, unweighted (Levitt, 1978)
LEVM780106	Normalized frequency of reverse turn, unweighted (Levitt, 1978)
LEWP710101	Frequency of occurrence in beta-bends (Lewis et al., 1971)
LIFS790101	Conformational preference for all beta-strands (Lifson-Sander, 1979)
LIFS790102	Conformational preference for parallel beta-strands (Lifson-Sander, 1979)
LIFS790103	Conformational preference for antiparallel beta-strands (Lifson-Sander, 1979)
MANP780101	Average surrounding hydrophobicity (Manavalan-Ponnuswamy, 1978)
MAXF760101	Normalized frequency of alpha-helix (Maxfield-Scheraga, 1976)
MAXF760102	Normalized frequency of extended structure (Maxfield-Scheraga, 1976)
MAXF760103	Normalized frequency of zeta R (Maxfield-Scheraga, 1976)
MAXF760104	Normalized frequency of left-handed alpha-helix (Maxfield-Scheraga, 1976)
MAXF760105	Normalized frequency of zeta L (Maxfield-Scheraga, 1976)
MAXF760106	Normalized frequency of alpha region (Maxfield-Scheraga, 1976)
MCMT640101	Refractivity (McMeekin et al., 1964), Cited by Jones (1975)
MEEJ800101	Retention coefficient in HPLC, pH7.4 (Meek, 1980)
MEEJ800102	Retention coefficient in HPLC, pH2.1 (Meek, 1980)
MEEJ810101	Retention coefficient in NaClO4 (Meek-Rossetti, 1981)
MEEJ810102	Retention coefficient in NaH2PO4 (Meek-Rossetti, 1981)
MEIH800101	Average reduced distance for C-alpha (Meirovitch et al., 1980)
MEIH800102	Average reduced distance for side chain (Meirovitch et al., 1980)
MEIH800103	Average side chain orientation angle (Meirovitch et al., 1980)
MIYS850101	Effective partition energy (Miyazawa-Jernigan, 1985)
NAGK730101	Normalized frequency of alpha-helix (Nagano, 1973)
NAGK730102	Normalized frequency of bata-structure (Nagano, 1973)
NAGK730103	Normalized frequency of coil (Nagano, 1973)
NAKH900101	AA composition of total proteins (Nakashima et al., 1990)
NAKH900102	SD of AA composition of total proteins (Nakashima et al., 1990)
NAKH900103	AA composition of mt-proteins (Nakashima et al., 1990)
NAKH900104	Normalized composition of mt-proteins (Nakashima et al., 1990)
NAKH900105	AA composition of mt-proteins from animal (Nakashima et al., 1990)

AAIndex	Description
NAKH900106	Normalized composition from animal (Nakashima et al., 1990)
NAKH900107	AA composition of mt-proteins from fungi and plant (Nakashima et al., 1990)
NAKH900108	Normalized composition from fungi and plant (Nakashima et al., 1990)
NAKH900109	AA composition of membrane proteins (Nakashima et al., 1990)
NAKH900110	Normalized composition of membrane proteins (Nakashima et al., 1990)
NAKH900111	Transmembrane regions of non-mt-proteins (Nakashima et al., 1990)
NAKH900112	Transmembrane regions of mt-proteins (Nakashima et al., 1990)
NAKH900113	Ratio of average and computed composition (Nakashima et al., 1990)
NAKH920101	AA composition of CYT of single-spanning proteins (Nakashima-Nishikawa, 1992)
NAKH920102	AA composition of CYT2 of single-spanning proteins (Nakashima-Nishikawa, 1992)
NAKH920103	AA composition of EXT of single-spanning proteins (Nakashima-Nishikawa, 1992)
NAKH920104	AA composition of EXT2 of single-spanning proteins (Nakashima-Nishikawa, 1992)
NAKH920105	AA composition of MEM of single-spanning proteins (Nakashima-Nishikawa, 1992)
NAKH920106	AA composition of CYT of multi-spanning proteins (Nakashima-Nishikawa, 1992)
NAKH920107	AA composition of EXT of multi-spanning proteins (Nakashima-Nishikawa, 1992)
NAKH920108	AA composition of MEM of multi-spanning proteins (Nakashima-Nishikawa, 1992)
NISK800101	8 A contact number (Nishikawa-Ooi, 1980)
NISK860101	14 A contact number (Nishikawa-Ooi, 1986)
NOZY710101	Transfer energy, organic solvent/water (Nozaki-Tanford, 1971)
OOBM770101	Average non-bonded energy per atom (Oobatake-Ooi, 1977)
OOBM770102	Short and medium range non-bonded energy per atom (Oobatake-Ooi, 1977)
OOBM770103	Long range non-bonded energy per atom (Oobatake-Ooi, 1977)
OOBM770104	Average non-bonded energy per residue (Oobatake-Ooi, 1977)
OOBM770105	Short and medium range non-bonded energy per residue (Oobatake-Ooi, 1977)
OOBM850101	Optimized beta-structure-coil equilibrium constant (Oobatake et al., 1985)
OOBM850102	Optimized propensity to form reverse turn (Oobatake et al., 1985)
OOBM850103	Optimized transfer energy parameter (Oobatake et al., 1985)
OOBM850104	Optimized average non-bonded energy per atom (Oobatake et al., 1985)
OOBM850105	Optimized side chain interaction parameter (Oobatake et al., 1985)
PALJ810101	Normalized frequency of alpha-helix from LG (Palau et al., 1981)
PALJ810102	Normalized frequency of alpha-helix from CF (Palau et al., 1981)
PALJ810103	Normalized frequency of beta-sheet from LG (Palau et al., 1981)
PALJ810104	Normalized frequency of beta-sheet from CF (Palau et al., 1981)
PALJ810105	Normalized frequency of turn from LG (Palau et al., 1981)
PALJ810106	Normalized frequency of turn from CF (Palau et al., 1981)
PALJ810107	Normalized frequency of alpha-helix in all-alpha class (Palau et al., 1981)
PALJ810108	Normalized frequency of alpha-helix in alpha+beta class (Palau et al., 1981)
PALJ810109	Normalized frequency of alpha-helix in alpha/beta class (Palau et al., 1981)
PALJ810110	Normalized frequency of beta-sheet in all-beta class (Palau et al., 1981)

AAIndex	Description
PALJ810111	Normalized frequency of beta-sheet in alpha+beta class (Palau et al., 1981)
PALJ810112	Normalized frequency of beta-sheet in alpha/beta class (Palau et al., 1981)
PALJ810113	Normalized frequency of turn in all-alpha class (Palau et al., 1981)
PALJ810114	Normalized frequency of turn in all-beta class (Palau et al., 1981)
PALJ810115	Normalized frequency of turn in alpha+beta class (Palau et al., 1981)
PALJ810116	Normalized frequency of turn in alpha/beta class (Palau et al., 1981)
PARJ860101	HPLC parameter (Parker et al., 1986)
PLIV810101	Partition coefficient (Pliska et al., 1981)
PONP800101	Surrounding hydrophobicity in folded form (Ponnuswamy et al., 1980)
PONP800102	Average gain in surrounding hydrophobicity (Ponnuswamy et al., 1980)
PONP800103	Average gain ratio in surrounding hydrophobicity (Ponnuswamy et al., 1980)
PONP800104	Surrounding hydrophobicity in alpha-helix (Ponnuswamy et al., 1980)
PONP800105	Surrounding hydrophobicity in beta-sheet (Ponnuswamy et al., 1980)
PONP800106	Surrounding hydrophobicity in turn (Ponnuswamy et al., 1980)
PONP800107	Accessibility reduction ratio (Ponnuswamy et al., 1980)
PONP800108	Average number of surrounding residues (Ponnuswamy et al., 1980)
PRAM820101	Intercept in regression analysis (Prabhakaran-Ponnuswamy, 1982)
PRAM820102	Slope in regression analysis x 1.0E1 (Prabhakaran-Ponnuswamy, 1982)
PRAM820103	Correlation coefficient in regression analysis (Prabhakaran-Ponnuswamy, 1982)
PRAM900101	Hydrophobicity (Prabhakaran, 1990)
PRAM900102	Relative frequency in alpha-helix (Prabhakaran, 1990)
PRAM900103	Relative frequency in beta-sheet (Prabhakaran, 1990)
PRAM900104	Relative frequency in reverse-turn (Prabhakaran, 1990)
PTIO830101	Helix-coil equilibrium constant (Ptitsyn-Finkelstein, 1983)
PTIO830102	Beta-coil equilibrium constant (Ptitsyn-Finkelstein, 1983)
QIAN880101	Weights for alpha-helix at the window position of -6 (Qian-Sejnowski, 1988)
QIAN880102	Weights for alpha-helix at the window position of -5 (Qian-Sejnowski, 1988)
QIAN880103	Weights for alpha-helix at the window position of -4 (Qian-Sejnowski, 1988)
QIAN880104	Weights for alpha-helix at the window position of -3 (Qian-Sejnowski, 1988)
QIAN880105	Weights for alpha-helix at the window position of -2 (Qian-Sejnowski, 1988)
QIAN880106	Weights for alpha-helix at the window position of -1 (Qian-Sejnowski, 1988)
QIAN880107	Weights for alpha-helix at the window position of 0 (Qian-Sejnowski, 1988)
QIAN880108	Weights for alpha-helix at the window position of 1 (Qian-Sejnowski, 1988)
QIAN880109	Weights for alpha-helix at the window position of 2 (Qian-Sejnowski, 1988)
QIAN880110	Weights for alpha-helix at the window position of 3 (Qian-Sejnowski, 1988)
QIAN880111	Weights for alpha-helix at the window position of 4 (Qian-Sejnowski, 1988)
QIAN880112	Weights for alpha-helix at the window position of 5 (Qian-Sejnowski, 1988)
QIAN880113	Weights for alpha-helix at the window position of 6 (Qian-Sejnowski, 1988)
QIAN880114	Weights for beta-sheet at the window position of -6 (Qian-Sejnowski, 1988)

AAIndex	Description
QIAN880115	Weights for beta-sheet at the window position of -5 (Qian-Sejnowski, 1988)
QIAN880116	Weights for beta-sheet at the window position of -4 (Qian-Sejnowski, 1988)
QIAN880117	Weights for beta-sheet at the window position of -3 (Qian-Sejnowski, 1988)
QIAN880118	Weights for beta-sheet at the window position of -2 (Qian-Sejnowski, 1988)
QIAN880119	Weights for beta-sheet at the window position of -1 (Qian-Sejnowski, 1988)
QIAN880120	Weights for beta-sheet at the window position of 0 (Qian-Sejnowski, 1988)
QIAN880121	Weights for beta-sheet at the window position of 1 (Qian-Sejnowski, 1988)
QIAN880122	Weights for beta-sheet at the window position of 2 (Qian-Sejnowski, 1988)
QIAN880123	Weights for beta-sheet at the window position of 3 (Qian-Sejnowski, 1988)
QIAN880124	Weights for beta-sheet at the window position of 4 (Qian-Sejnowski, 1988)
QIAN880125	Weights for beta-sheet at the window position of 5 (Qian-Sejnowski, 1988)
QIAN880126	Weights for beta-sheet at the window position of 6 (Qian-Sejnowski, 1988)
QIAN880127	Weights for coil at the window position of -6 (Qian-Sejnowski, 1988)
QIAN880128	Weights for coil at the window position of -5 (Qian-Sejnowski, 1988)
QIAN880129	Weights for coil at the window position of -4 (Qian-Sejnowski, 1988)
QIAN880130	Weights for coil at the window position of -3 (Qian-Sejnowski, 1988)
QIAN880131	Weights for coil at the window position of -2 (Qian-Sejnowski, 1988)
QIAN880132	Weights for coil at the window position of -1 (Qian-Sejnowski, 1988)
QIAN880133	Weights for coil at the window position of 0 (Qian-Sejnowski, 1988)
QIAN880134	Weights for coil at the window position of 1 (Qian-Sejnowski, 1988)
QIAN880135	Weights for coil at the window position of 2 (Qian-Sejnowski, 1988)
QIAN880136	Weights for coil at the window position of 3 (Qian-Sejnowski, 1988)
QIAN880137	Weights for coil at the window position of 4 (Qian-Sejnowski, 1988)
QIAN880138	Weights for coil at the window position of 5 (Qian-Sejnowski, 1988)
QIAN880139	Weights for coil at the window position of 6 (Qian-Sejnowski, 1988)
RACS770101	Average reduced distance for C-alpha (Rackovsky-Scheraga, 1977)
RACS770102	Average reduced distance for side chain (Rackovsky-Scheraga, 1977)
RACS770103	Side chain orientational preference (Rackovsky-Scheraga, 1977)
RACS820101	Average relative fractional occurrence in A0(i) (Rackovsky-Scheraga, 1982)
RACS820102	Average relative fractional occurrence in AR(i) (Rackovsky-Scheraga, 1982)
RACS820103	Average relative fractional occurrence in AL(i) (Rackovsky-Scheraga, 1982)
RACS820104	Average relative fractional occurrence in EL(i) (Rackovsky-Scheraga, 1982)
RACS820105	Average relative fractional occurrence in E0(i) (Rackovsky-Scheraga, 1982)
RACS820106	Average relative fractional occurrence in ER(i) (Rackovsky-Scheraga, 1982)
RACS820107	Average relative fractional occurrence in A0(i-1) (Rackovsky-Scheraga, 1982)
RACS820108	Average relative fractional occurrence in AR(i-1) (Rackovsky-Scheraga, 1982)
RACS820109	Average relative fractional occurrence in AL(i-1) (Rackovsky-Scheraga, 1982)
RACS820110	Average relative fractional occurrence in EL(i-1) (Rackovsky-Scheraga, 1982)
RACS820111	Average relative fractional occurrence in E0(i-1) (Rackovsky-Scheraga, 1982)

AAIndex	Description
RACS820112	Average relative fractional occurrence in ER(i-1) (Rackovsky-Scheraga, 1982)
RACS820113	Value of theta(i) (Rackovsky-Scheraga, 1982)
RACS820114	Value of theta(i-1) (Rackovsky-Scheraga, 1982)
RADA880101	Transfer free energy from chx to wat (Radzicka-Wolfenden, 1988)
RADA880102	Transfer free energy from oct to wat (Radzicka-Wolfenden, 1988)
RADA880103	Transfer free energy from vap to chx (Radzicka-Wolfenden, 1988)
RADA880104	Transfer free energy from chx to oct (Radzicka-Wolfenden, 1988)
RADA880105	Transfer free energy from vap to oct (Radzicka-Wolfenden, 1988)
RADA880106	Accessible surface area (Radzicka-Wolfenden, 1988)
RADA880107	Energy transfer from out to in(95%buried) (Radzicka-Wolfenden, 1988)
RADA880108	Mean polarity (Radzicka-Wolfenden, 1988)
RICJ880101	Relative preference value at N" (Richardson-Richardson, 1988)
RICJ880102	Relative preference value at N' (Richardson-Richardson, 1988)
RICJ880103	Relative preference value at N-cap (Richardson-Richardson, 1988)
RICJ880104	Relative preference value at N1 (Richardson-Richardson, 1988)
RICJ880105	Relative preference value at N2 (Richardson-Richardson, 1988)
RICJ880106	Relative preference value at N3 (Richardson-Richardson, 1988)
RICJ880107	Relative preference value at N4 (Richardson-Richardson, 1988)
RICJ880108	Relative preference value at N5 (Richardson-Richardson, 1988)
RICJ880109	Relative preference value at Mid (Richardson-Richardson, 1988)
RICJ880110	Relative preference value at C5 (Richardson-Richardson, 1988)
RICJ880111	Relative preference value at C4 (Richardson-Richardson, 1988)
RICJ880112	Relative preference value at C3 (Richardson-Richardson, 1988)
RICJ880113	Relative preference value at C2 (Richardson-Richardson, 1988)
RICJ880114	Relative preference value at C1 (Richardson-Richardson, 1988)
RICJ880115	Relative preference value at C-cap (Richardson-Richardson, 1988)
RICJ880116	Relative preference value at C' (Richardson-Richardson, 1988)
RICJ880117	Relative preference value at C" (Richardson-Richardson, 1988)
ROBB760101	Information measure for alpha-helix (Robson-Suzuki, 1976)
ROBB760102	Information measure for N-terminal helix (Robson-Suzuki, 1976)
ROBB760103	Information measure for middle helix (Robson-Suzuki, 1976)
ROBB760104	Information measure for C-terminal helix (Robson-Suzuki, 1976)
ROBB760105	Information measure for extended (Robson-Suzuki, 1976)
ROBB760106	Information measure for pleated-sheet (Robson-Suzuki, 1976)
ROBB760107	Information measure for extended without H-bond (Robson-Suzuki, 1976)
ROBB760108	Information measure for turn (Robson-Suzuki, 1976)
ROBB760109	Information measure for N-terminal turn (Robson-Suzuki, 1976)
ROBB760110	Information measure for middle turn (Robson-Suzuki, 1976)
ROBB760111	Information measure for C-terminal turn (Robson-Suzuki, 1976)

AAIndex	Description
ROBB760112	Information measure for coil (Robson-Suzuki, 1976)
ROBB760113	Information measure for loop (Robson-Suzuki, 1976)
ROBB790101	Hydration free energy (Robson-Osguthorpe, 1979)
ROSG850101	Mean area buried on transfer (Rose et al., 1985)
ROSG850102	Mean fractional area loss (Rose et al., 1985)
ROSM880101	Side chain hydropathy, uncorrected for solvation (Roseman, 1988)
ROSM880102	Side chain hydropathy, corrected for solvation (Roseman, 1988)
ROSM880103	Loss of Side chain hydropathy by helix formation (Roseman, 1988)
SIMZ760101	Transfer free energy (Simon, 1976), Cited by Charton-Charton (1982)
SNEP660101	Principal component I (Sneath, 1966)
SNEP660102	Principal component II (Sneath, 1966)
SNEP660103	Principal component III (Sneath, 1966)
SNEP660104	Principal component IV (Sneath, 1966)
SUEM840101	Zimm-Bragg parameter s at 20 C (Sueki et al., 1984)
SUEM840102	Zimm-Bragg parameter sigma x 1.0E4 (Sueki et al., 1984)
SWER830101	Optimal matching hydrophobicity (Sweet-Eisenberg, 1983)
TANS770101	Normalized frequency of alpha-helix (Tanaka-Scheraga, 1977)
TANS770102	Normalized frequency of isolated helix (Tanaka-Scheraga, 1977)
TANS770103	Normalized frequency of extended structure (Tanaka-Scheraga, 1977)
TANS770104	Normalized frequency of chain reversal R (Tanaka-Scheraga, 1977)
TANS770105	Normalized frequency of chain reversal S (Tanaka-Scheraga, 1977)
TANS770106	Normalized frequency of chain reversal D (Tanaka-Scheraga, 1977)
TANS770107	Normalized frequency of left-handed helix (Tanaka-Scheraga, 1977)
TANS770108	Normalized frequency of zeta R (Tanaka-Scheraga, 1977)
TANS770109	Normalized frequency of coil (Tanaka-Scheraga, 1977)
TANS770110	Normalized frequency of chain reversal (Tanaka-Scheraga, 1977)
VASM830101	Relative population of conformational state A (Vasquez et al., 1983)
VASM830102	Relative population of conformational state C (Vasquez et al., 1983)
VASM830103	Relative population of conformational state E (Vasquez et al., 1983)
VELV850101	Electron-ion interaction potential (Veljkovic et al., 1985)
VENT840101	Bitterness (Venanzi, 1984)
VHEG790101	Transfer free energy to lipophilic phase (von Heijne-Blomberg, 1979)
WARP780101	Average interactions per side chain atom (Warme-Morgan, 1978)
WEBA780101	RF value in high salt chromatography (Weber-Lacey, 1978)
WERD780101	Propensity to be buried inside (Wertz-Scheraga, 1978)
WERD780102	Free energy change of epsilon(i) to epsilon(ex) (Wertz-Scheraga, 1978)
WERD780103	Free energy change of alpha(Ri) to alpha(Rh) (Wertz-Scheraga, 1978)
WERD780104	Free energy change of epsilon(i) to alpha(Rh) (Wertz-Scheraga, 1978)
WOEC730101	Polar requirement (Woese, 1973)

AAIndex	Description
WOLR810101	Hydration potential (Wolfenden et al., 1981)
WOLS870101	Principal property value z1 (Wold et al., 1987)
WOLS870102	Principal property value z2 (Wold et al., 1987)
WOLS870103	Principal property value z3 (Wold et al., 1987)
YUTK870101	Unfolding Gibbs energy in water, pH7.0 (Yutani et al., 1987)
YUTK870102	Unfolding Gibbs energy in water, pH9.0 (Yutani et al., 1987)
YUTK870103	Activation Gibbs energy of unfolding, pH7.0 (Yutani et al., 1987)
YUTK870104	Activation Gibbs energy of unfolding, pH9.0 (Yutani et al., 1987)
ZASB820101	Dependence of partition coefficient on ionic strength (Zaslavsky et al., 1982)
ZIMJ680101	Hydrophobicity (Zimmerman et al., 1968)
ZIMJ680102	Bulkiness (Zimmerman et al., 1968)
ZIMJ680103	Polarity (Zimmerman et al., 1968)
ZIMJ680104	Isoelectric point (Zimmerman et al., 1968)
ZIMJ680105	RF rank (Zimmerman et al., 1968)
AURR980101	Normalized positional residue frequency at helix termini N4'(Aurora-Rose, 1998)
AURR980102	Normalized positional residue frequency at helix termini N"' (Aurora-Rose, 1998)
AURR980103	Normalized positional residue frequency at helix termini N" (Aurora-Rose, 1998)
AURR980104	Normalized positional residue frequency at helix termini N'(Aurora-Rose, 1998)
AURR980105	Normalized positional residue frequency at helix termini Nc (Aurora-Rose, 1998)
AURR980106	Normalized positional residue frequency at helix termini N1 (Aurora-Rose, 1998)
AURR980107	Normalized positional residue frequency at helix termini N2 (Aurora-Rose, 1998)
AURR980108	Normalized positional residue frequency at helix termini N3 (Aurora-Rose, 1998)
AURR980109	Normalized positional residue frequency at helix termini N4 (Aurora-Rose, 1998)
AURR980110	Normalized positional residue frequency at helix termini N5 (Aurora-Rose, 1998)
AURR980111	Normalized positional residue frequency at helix termini C5 (Aurora-Rose, 1998)
AURR980112	Normalized positional residue frequency at helix termini C4 (Aurora-Rose, 1998)
AURR980113	Normalized positional residue frequency at helix termini C3 (Aurora-Rose, 1998)
AURR980114	Normalized positional residue frequency at helix termini C2 (Aurora-Rose, 1998)
AURR980115	Normalized positional residue frequency at helix termini C1 (Aurora-Rose, 1998)
AURR980116	Normalized positional residue frequency at helix termini Cc (Aurora-Rose, 1998)
AURR980117	Normalized positional residue frequency at helix termini C' (Aurora-Rose, 1998)
AURR980118	Normalized positional residue frequency at helix termini C" (Aurora-Rose, 1998)
AURR980119	Normalized positional residue frequency at helix termini C"' (Aurora-Rose, 1998)
AURR980120	Normalized positional residue frequency at helix termini C4' (Aurora-Rose, 1998)
ONEK900101	Delta G values for the peptides extrapolated to 0 M urea (O'Neil-DeGrado, 1990)
ONEK900102	Helix formation parameters (delta delta G) (O'Neil-DeGrado, 1990)
VINM940101	Normalized flexibility parameters (B-values), average (Vihinen et al., 1994)
VINM940102	Normalized flexibility parameters (B-values) for each residue surrounded by none rigid neighbours (Vihinen et al., 1994)

AAIndex	Description
VINM940103	Normalized flexibility parameters (B-values) for each residue surrounded by one rigid neighbours (Vihinen et al., 1994)
VINM940104	Normalized flexibility parameters (B-values) for each residue surrounded by two rigid neighbours (Vihinen et al., 1994)
MUNV940101	Free energy in alpha-helical conformation (Munoz-Serrano, 1994)
MUNV940102	Free energy in alpha-helical region (Munoz-Serrano, 1994)
MUNV940103	Free energy in beta-strand conformation (Munoz-Serrano, 1994)
MUNV940104	Free energy in beta-strand region (Munoz-Serrano, 1994)
MUNV940105	Free energy in beta-strand region (Munoz-Serrano, 1994)
WIMW960101	Free energies of transfer of AcWl-X-LL peptides from bilayer interface to water (Wimley-White, 1996)
KIMC930101	Thermodynamic beta sheet propensity (Kim-Berg, 1993)
MONM990101	Turn propensity scale for transmembrane helices (Monne et al., 1999)
BLAM930101	Alpha helix propensity of position 44 in T4 lysozyme (Blaber et al., 1993)
PARS000101	p-Values of mesophilic proteins based on the distributions of B values (Parthasarathy-Murthy, 2000)
PARS000102	p-Values of thermophilic proteins based on the distributions of B values (Parthasarathy-Murthy, 2000)
KUMS000101	Distribution of amino acid residues in the 18 non-redundant families of thermophilic proteins (Kumar et al., 2000)
KUMS000102	Distribution of amino acid residues in the 18 non-redundant families of mesophilic proteins (Kumar et al., 2000)
KUMS000103	Distribution of amino acid residues in the alpha-helices in thermophilic proteins (Kumar et al., 2000)
KUMS000104	Distribution of amino acid residues in the alpha-helices in mesophilic proteins (Kumar et al., 2000)
TAKK010101	Side-chain contribution to protein stability (kJ/mol) (Takano-Yutani, 2001)
FODM020101	Propensity of amino acids within pi-helices (Fodje-Al-Karadaghi, 2002)
NADH010101	Hydropathy scale based on self-information values in the two-state model (5 accessibility) (Naderi-Manesh et al., 2001)
NADH010102	Hydropathy scale based on self-information values in the two-state model (9 accessibility) (Naderi-Manesh et al., 2001)
NADH010103	Hydropathy scale based on self-information values in the two-state model (16 accessibility) (Naderi-Manesh et al., 2001)
NADH010104	Hydropathy scale based on self-information values in the two-state model (20 accessibility) (Naderi-Manesh et al., 2001)
NADH010105	Hydropathy scale based on self-information values in the two-state model (25 accessibility) (Naderi-Manesh et al., 2001)
NADH010106	Hydropathy scale based on self-information values in the two-state model

AAIndex	Description
	(36 accessibility) (Naderi-Manesh et al., 2001)
NADH010107	Hydropathy scale based on self-information values in the two-state model (50 accessibility) (Naderi-Manesh et al., 2001)
MONM990201	Averaged turn propensities in a transmembrane helix (Monne et al., 1999)
KOEP990101	Alpha-helix propensity derived from designed sequences (Koehl-Levitt, 1999)
KOEP990102	Beta-sheet propensity derived from designed sequences (Koehl-Levitt, 1999)
CEDJ970101	Composition of amino acids in extracellular proteins (percent) (Cedano et al., 1997)
CEDJ970102	Composition of amino acids in anchored proteins (percent) (Cedano et al., 1997)
CEDJ970103	Composition of amino acids in membrane proteins (percent) (Cedano et al., 1997)
CEDJ970104	Composition of amino acids in intracellular proteins (percent) (Cedano et al., 1997)
CEDJ970105	Composition of amino acids in nuclear proteins (percent) (Cedano et al., 1997)
FUKS010101	Surface composition of amino acids in intracellular proteins of thermophiles (percent) (Fukuchi-Nishikawa, 2001)
FUKS010102	Surface composition of amino acids in intracellular proteins of mesophiles (percent) (Fukuchi-Nishikawa, 2001)
FUKS010103	Surface composition of amino acids in extracellular proteins of mesophiles (percent) (Fukuchi-Nishikawa, 2001)
FUKS010104	Surface composition of amino acids in nuclear proteins (percent) (Fukuchi-Nishikawa, 2001)
FUKS010105	Interior composition of amino acids in intracellular proteins of thermophiles (percent) (Fukuchi-Nishikawa, 2001)
FUKS010106	Interior composition of amino acids in intracellular proteins of mesophiles (percent) (Fukuchi-Nishikawa, 2001)
FUKS010107	Interior composition of amino acids in extracellular proteins of mesophiles (percent) (Fukuchi-Nishikawa, 2001)
FUKS010108	Interior composition of amino acids in nuclear proteins (percent) (Fukuchi-Nishikawa, 2001)
FUKS010109	Entire chain composition of amino acids in intracellular proteins of thermophiles (percent) (Fukuchi-Nishikawa, 2001)
FUKS010110	Entire chain composition of amino acids in intracellular proteins of mesophiles (percent) (Fukuchi-Nishikawa, 2001)
FUKS010111	Entire chain composition of amino acids in extracellular proteins of mesophiles (percent) (Fukuchi-Nishikawa, 2001)
FUKS010112	Entire chain compositino of amino acids in nuclear proteins (percent) (Fukuchi-Nishikawa, 2001)
MITS020101	Amphiphilicity index (Mitaku et al., 2002)
TSAJ990101	Volumes including the crystallographic waters using the ProtOr (Tsai et al., 1999)
TSAJ990102	Volumes not including the crystallographic waters using the ProtOr (Tsai et al., 1999)
COSI940101	Electron-ion interaction potential values (Cosic, 1994)
PONP930101	Hydrophobicity scales (Ponnuswamy, 1993)
WILM950101	Hydrophobicity coefficient in RP-HPLC, C18 with 0.1%TFA/MeCN/H2O (Wilce et al. 1995)
WILM950102	Hydrophobicity coefficient in RP-HPLC, C8 with 0.1%TFA/MeCN/H2O (Wilce et al. 1995)
WILM950103	Hydrophobicity coefficient in RP-HPLC, C4 with 0.1%TFA/MeCN/H2O (Wilce et al. 1995)
WILM950104	Hydrophobicity coefficient in RP-HPLC, C18 with 0.1%TFA/2-PrOH/MeCN/H2O (Wilce et al. 1995)
KUHL950101	Hydrophilicity scale (Kuhn et al., 1995)
GUOD860101	Retention coefficient at pH 2 (Guo et al., 1986)
JURD980101	Modified Kyte-Doolittle hydrophobicity scale (Juretic et al., 1998)
